# Influence of Sex and Sex-Based Disparities on Prevalent Tuberculosis, Vietnam, 2017–2018

**DOI:** 10.3201/eid2905.221476

**Published:** 2023-05

**Authors:** Hai Viet Nguyen, Daniella Brals, Edine Tiemersma, Robert Gasior, Nhung Viet Nguyen, Hoa Binh Nguyen, Hung Van Nguyen, Ngoc Anh Le Thi, Frank Cobelens

**Affiliations:** Amsterdam University Medical Centers, University of Amsterdam, Amsterdam, the Netherlands (H.V. Nguyen, D. Brals, F. Cobelens);; Vietnam National Tuberculosis Program, Hanoi, Vietnam (H.V. Nguyen, N.V. Nguyen, H.B. Nguyen, H.V. Nguyen, N.A. Le Thi);; KNCV Tuberculosis Foundation, Den Haag, the Netherlands (E. Tiemersma);; National Academies of Sciences, Engineering, and Medicine, Washington, DC, USA (R. Gasior).

**Keywords:** tuberculosis and other mycobacteria, bacteria, Vietnam, sex-based disparities, nested case-control study, epidemiology

## Abstract

To assess sex disparities in tuberculosis in Vietnam, we conducted a nested, case–control study based on a 2017 tuberculosis prevalence survey. We defined the case group as all survey participants with laboratory–confirmed tuberculosis and the control group as a randomly selected group of participants with no tuberculosis. We used structural equation modeling to describe pathways from sex to tuberculosis according to an a priori conceptual framework. Our analysis included 1,319 participants, of whom 250 were case-patients. We found that sex was directly associated with tuberculosis prevalence (adjusted odds ratio for men compared with women 3.0 [95% CI 1.7–5.0]) and indirectly associated through other domains. The strong sex difference in tuberculosis prevalence is explained by a complex interplay of factors relating to behavioral and environmental risks, access to healthcare, and clinical manifestations. However, after controlling for all those factors, a direct sex effect remains that might be caused by biological factors.

Tuberculosis (TB) affects millions of persons worldwide. In most regions, the TB notification rate for men is higher than for women. According to the World Health Organization, the male-to-female (M:F) ratio of notified TB cases in 2020 was 1.7 globally; 56% of all TB cases were in men, 33% in women, and 11% in children (*1*). Several studies indicate that the high M:F ratio is mainly because cases in women are undernotified and because of bias in case reporting (*2*,*3*). However, a meta-analysis of 56 surveys of TB prevalence around the world showed a M:F ratio of 2.2, with the highest ratio in Southeast Asia (3.4, 95% CI 2.8–4.0) (*4*), suggesting that the sex variation in TB exists independently of reporting. Commonly proposed reasons behind this phenomenon include differences in how men and women seek and access TB care, how social roles affect contact with persons harboring *Mycobacterium tuberculosis* infection, and how certain male-dominated occupations (e.g., mining) can increase the risk for TB (*4*). Moreover, risk factors for TB, such as tobacco smoking and excessive alcohol consumption, are generally more frequent among men (*5*). Some studies have suggested biological differences in TB risk between men and women (*6*). The relative contributions of those factors in explaining the differences in TB risk between men and women are still under debate, and they may confound or obscure one another.

Vietnam, 1 of 30 countries that carries a high TB burden, conducted a national TB prevalence survey in 2017, coordinated by its National TB Program (*7*). That survey found a TB prevalence of 322 (95% CI 260–399) cases/100,000 adults nationally and a M:F ratio of 4.0. We conducted a nested, case–control study within this TB prevalence survey to assess the contribution of various factors that might have contributed to that ratio, including access to health care, exposure, socioeconomic status (SES), and possible biological factors. We acknowledge that other potentially underlying sex disparities could also have contributed.

## Methods

### Study Population

The second national TB prevalence survey in Vietnam was conducted during October 2017–February 2018, using multistage cluster sampling to select 87,207 eligible participants ≥15 years of age from 82 population clusters across the country (*7*). Using this survey, we designed a nested, case–control study (PEER study) in which the case group consisted of all participants with >1 positive TB test (Xpert MTB/Rif; Cepeheid, https://www.cepheid.com) conducted in the field. The control group consisted of persons who screened negative for TB or had an Xpert-negative result. We interviewed participants using an in-depth questionnaire to assess their TB-suggestive symptoms, access to health care, and TB-associated risk factors.

### Case Group Selection

We defined a case-patient as an eligible participant who had >1 positive Xpert result. To identify case-patients, we screened all survey participants for TB by a short questionnaire and chest radiograph. We defined presumptive TB cases as persons who had cough for >2 weeks (or cough of any duration for pregnant women), self-reported TB treatment in the 2 years preceding the survey, or chest radiograph with abnormalities consistent with TB; we asked those persons to provide a sputum sample. We collected the first sputum sample at the initial screening and examined it with Xpert either in the district laboratory or directly in the field. If the Xpert result was positive for M*. tuberculosis*, we asked the participants to provide an additional sputum sample the next day for confirmation, invited them to participate in the PEER study, and interviewed them after collecting consent on submission of the second sample.

### Control Group Selection

We defined a control as an eligible participant who was randomly selected for the PEER study, attended the screening event and screened negative for TB, or had a negative Xpert result. Before the TB screening event started, we conducted a house-to-house census in each cluster. All eligible adults received an invitation card with a unique PIN code. We randomly selected the PIN code of participants in each cluster to be recruited in the control group, regardless of the number of TB cases detected in each cluster. We invited those who attended the screening event to participate in the PEER study. Trained interviewers interviewed persons consenting to participate.

### Sample Size

On the basis of TB prevalence in Vietnam in 2007 and the assumed decline in TB burden (*8*,*9*), we expected to find 3 TB cases per cluster, resulting in 246 cases. Per case, aiming to achieve a final case-to-control ratio of 1:4, we randomly selected 5 controls to account for persons refusing participation and loss of controls because of Xpert-positive results. This process resulted in a control group sample size of 1,230 participants.

### Data Analysis

**Figure 1:**
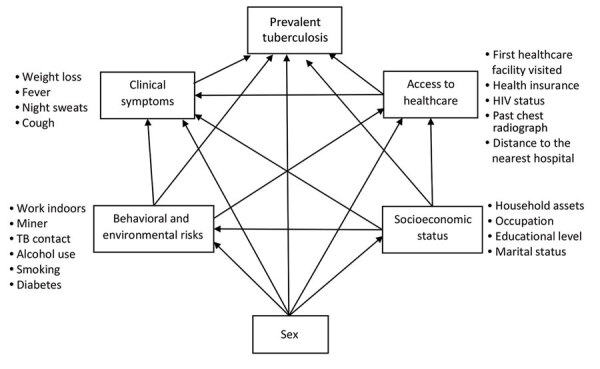
Conceptual framework of the structural equation model to describe pathways between tuberculosis prevalence, sex, and the associated domains for case–control analysis of tuberculosis prevalence, Vietnam, 2017–2018.

We used structural equation modeling (SEM) to describe pathways to prevalent TB through sex and gender-related exposures according to an a priori conceptual framework (Figure 1) (*10*). In addition to the main exposure, sex, we examined exposures grouped a priori into pre-conceived domains (i.e., SES, access to healthcare, TB behavioral and environmental risks, and clinical symptoms), reflecting factors likely to influence the risk for TB disease or affect TB disease detection (*11*–*14*). Because we analyzed prevalence of disease rather than incidence, and the relationship between prevalence and incidence (i.e., the duration of TB disease) is affected by disease detection, we added clinical symptoms and access to healthcare to our analysis to address this potential selection bias. For each preconceived domain, we performed confirmatory factor analysis where we estimated the domain score as a stand-alone latent variable using all relevant underlying variables collected from the in-depth questionnaire and household visits (Figure 1; Appendix Table 1). The clinical symptoms domain consisted of weight loss, fever, night sweats, and cough. The access to healthcare domain consisted of health insurance status, HIV status, previous chest radiograph, types of healthcare facilities visited due to reported symptoms, and distance from the nearest hospital. The behavioral and environmental risks domain consisted of working indoors or as a miner, being close contacts of TB patients, having diabetes, excessive drinking of alcoholic beverages, and tobacco smoking (including the amount of pack-years and passive smoking). The SES domain consisted of household assets, participants’ occupations, marital status, and level of education. 

**Table 1:** Characteristics of participants (N = 1,319) in case–control analysis of tuberculosis prevalence, Vietnam, 2017–2018

Characteristics	Controls, no. (%)	Cases, no. (%)	Total, no. (%)
Sex			
M	493 (46.1)	201 (80.4)	694 (52.6)
F	576 (53.9)	49 (19.6)	625 (47.4)
Age groups, y			
15–24	77 (7.2)	7 (2.8)	84 (6.4)
25–34	193 (18.0)	25 (10.0)	218 (16.5)
35–44	216 (20.2)	33 (13.2)	249 (18.9)
45–54	239 (22.4)	62 (24.8)	301 (22.8)
55–64	212 (19.8)	61 (24.4)	273 (20.7)
≥65	132 (12.4)	62 (24.8)	194 (14.7)
Area			
Urban	447 (41.8)	96 (38.4)	543 (41.2)
Remote	255 (23.9)	59 (23.6)	314 (23.8)
Rural	367 (34.3)	95 (38.0)	462 (35.0)
Region			
North	438 (41.0)	74 (29.6)	512 (38.8)
Center	190 (17.8)	41 (16.4)	231 (17.5)
South	441 (41.2)	135 (54.0)	576 (43.7)
Clinical symptoms			
Least severe	760 (71.1)	71 (28.4)	831 (63.0)
Moderately severe	245 (22.9)	112 (44.8)	357 (27.1)
Most severe	64 (6.0)	67 (26.8)	131 (9.9)
Access to healthcare			
Good	451 (42.2)	20 (8.0)	471 (35.7)
Moderate	379 (35.4)	49 (19.6)	428 (32.5)
Poor	239 (22.4)	181 (72.4)	420 (31.8)
Behavioral and environmental risk			
Low	461 (43.1)	34 (13.6)	495 (37.5)
Moderate	358 (33.5)	76 (30.4)	431 (32.7)
High	250 (23.4)	140 (56.2)	393 (29.8)
Socioeconomic status			
High	389 (34.0)	60 (24.0)	609 (46.2)
Medium	373 (33.2)	65 (26.0)	334 (25.3)
Low	307 (32.8)	125 (50.0)	376 (28.5)
Total	1,069 (100)	250 (100)	1,319 (100)

We computed a predicted latent continuous variable for each domain. Using the xtile command in Stata (StataCorp LLC, https://www.stata.com), we categorized each latent domain variable into relatively equal tertiles that represent the highest to lowest values of the latent variables (*15*). For the clinical symptoms domain, the tertiles were least severe, moderately severe, and most severe; for the access to healthcare domain, they were good access, moderate access, and poor access; for the behavioral and environmental risk domain, they were low risk, moderate risk, and high risk; and for the SES domain, they were high, medium, and low.

To investigate the pathways from sex to TB, we included the domain tertile scores in a multivariable SEM according to the conceptual framework (Figure 1). We modeled TB within the SEM using the Bernoulli logit model, and we fit the predicted latent domain variables using ordinal logit models. We adjusted each outcome within the SEM for age, area (urban, remote, and rural), and region (North, Central, or South). In reporting, we distinguished direct pathways (from a domain to an outcome) and indirect pathways (from a domain to an outcome via another domain). We assessed goodness-of-fit by using bootstrapped area under the curve (1,000 replications). We employed Stata version 14.0 for statistical analyses.

We included the clinical symptoms domain in our conceptual framework because having symptoms increases the chance for TB patients to be identified through the survey process but also to be diagnosed with TB during routine care before the survey, thereby controlling for potential selection bias inherent in a prevalence survey. We acknowledged that doing so might obscure the effects of other domains by being on the causal pathway between sex and prevalent TB. In the TB prevalence survey, the final status of a TB case was decided by an expert panel, not just by the result of Xpert. We therefore performed sensitivity analyses in which the clinical domain was excluded (model 1), the definition of a TB case was replaced with the expert panel decision (model 2), and Xpert-positive TB cases with previous TB treatment history were excluded (model 3). We applied inverse probability weighting and poststratification weighting to adjust for differences in participation rate by age, sex, cluster, and the relative contribution of each participant, as described in our previous publication (*7*).

### Ethics Statement

This study was given scientific and ethical approval by the Institutional Review Board of the Vietnam National Lung Hospital, under approval letter number 62/17/CTHĐKH-ĐĐ. All participants signed an individual written informed consent. All of those in the case group were referred to their local district TB unit for appropriate treatment.

## Results

**Figure 2:**
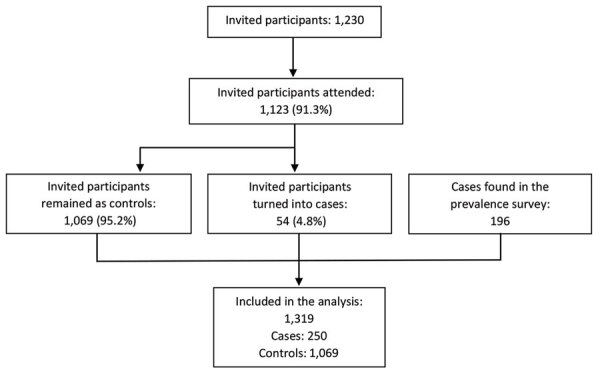
Summary of the data flow for case–control analysis of tuberculosis prevalence, Vietnam, 2017–2018.

Of the 1,230 preselected controls, 107 did not attend the screening event or declined to participate, and 54 had a positive Xpert result that defined them as TB cases (Figure 2). There were 1,319 interviewed participants in total, of which 250 were cases and 1,069 were controls (Table 1); 694 (52.6%) participants were men and 625 (47.4%) women. Among the controls, 2 were later reported to have positive culture results, classified as TB cases by the expert panel, and excluded from our analysis. 

The distribution of men and women among the domains (Table 2) shows that men were more dominant in the most disadvantaged tertile of all domains, except for SES. Compared with women, men more often reported severe clinical symptoms (12.8% vs. 6.7%), poor access to healthcare (38.9% vs. 24.0%), and high behavioral and environmental risk (52.9% vs. 4.2%) (Table 1).

**Table 2:** Distribution of domains by sex among interviewed participants (N = 1,319) in case–control analysis of tuberculosis prevalence, Vietnam, 2017–2018

Domains	Women, no. (%)	Men, no. (%)	Total, no. (%)	p value*
Clinical symptoms				
Least	439 (70.2)	392 (56.5)	831 (63.0)	<0.001
Moderately	144 (23.1)	213 (30.7)	357 (27.1)	
Most	42 (6.7)	89 (12.8)	131 (9.9)	
Access to healthcare				
Good	256 (41.0)	215 (31.0)	471 (35.7)	<0.001
Moderate	219 (35.0)	209 (30.1)	428 (32.5)	
Poor	150 (24.0)	270 (38.9)	420 (31.8)	
Behavioral and environmental risk				
Low	347 (55.5)	148 (21.3)	495 (37.5)	<0.001
Moderate	252 (40.3)	182 (26.2)	434 (32.9)	
High	26 (4.2)	364 (52.5)	390 (29.6)	
Socioeconomic status				
High	202 (32.3)	247 (35.6)	449 (34.0)	0.440
Medium	215 (34.4)	223 (32.1)	438 (33.2)	
Low	208 (33.3)	224 (32.3)	432 (32.8)	
Total	625 (100)	694 (100)	1,319 (100)	

*By Pearson χ^2^ test to compare the differences between men and women.

**Figure 3:**
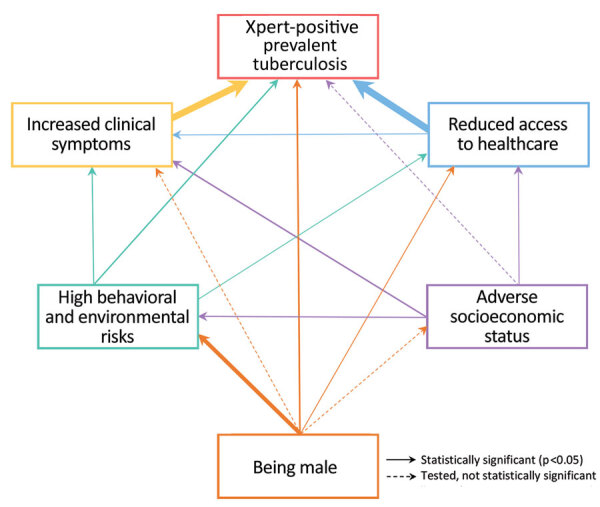
Structural equation model of the relationships between domains and Xpert-positive (Xpert MTB/Rif; Cepeheid, https://www.cepheid.com) tuberculosis prevalence for case–control analysis of tuberculosis prevalence, Vietnam, 2017–2018. For significant associations, the arrow thickness corresponds to the effect size. Each outcome was adjusted for age, area, and region. See Table 3 for the full estimation results. Model results were weighted using sampling and lost-to-follow-up weights. Bootstrapped area under the curve (1,000 replications) was 0.90 (95% CI 0.89–0.92).

SEM results (Figure 3), full estimation results (Table 3), and the confirmatory factor analysis results (Appendix Table 1) indicate that sex was directly associated with prevalent TB (adjusted odds ratio [aOR] for men compared with women 3.0 [95% CI 1.7–5.0]). Sex was also associated indirectly with prevalent TB through behavioral and environmental risks, behavioral and environmental risks and clinical symptoms, and behavioral and environmental risks and access to healthcare. Clinical symptoms (aOR for moderate severity 5.0 [95% CI 2.8–8.7]; aOR for most severe 13.2 [95% CI 5.9–29.2]), access to healthcare (aOR for moderate access 2.5 [95% CI 1.4–4.4]; aOR for poor access 13.4 [95% CI 7.0–25.6]), and behavioral and environmental risks (aOR for moderate risk 2.1 [95% CI 1.1–3.9]; aOR for high risk 2.9 [95% CI 1.5–5.6]) were directly associated with TB as well. SES did not directly affect prevalent TB, only indirectly through access to healthcare, behavioral and environmental risks, and clinical symptoms (Figure 3, Table 3). When we stratified the SEM model by sex, we found a statistically significant association between prevalent TB and behavioral and environmental risks among men (aOR for moderate risk 3.5 [95% CI 1.5–8.2]; aOR for high risk 4.8 (95% CI 2.0–11.7]). That same association was not statistically significant among women (Appendix Figures 1 and 2).

**Table 3:** Structural equation model full estimation results of the relationships between sex, clinical symptoms, behavioral and environmental risks, access to healthcare, SES, and Xpert-positive prevalent tuberculosis, Vietnam, 2017–2018*

Outcomes	Xpert-postive prevalent TB			Clinical symptoms			Access to healthcare			Behavioral and environmental risks			Socioeconomic status	
aOR (95% CI)	p value	aOR (95% CI)	p value	aOR (95% CI)	p value	aOR (95% CI)	p value	aOR (95% CI)	p value
Male sex	3.0 (1.7–5.0)	<0.001		1.2 (0.9–1.7)	0.281		1.4 (1.1–1.7)	0.005		7.7 (6.0–9.8)	<0.001		0.8 (0.6–1.0)	0.172
Age groups, y														
15–24	Referent			Referent			Referent			Referent			Referent	
25–34	1.1 (0.4–2.8)	0.824		1.2 (0.5–2.4)	0.706		1.1 (0.7–1.7)	0.611		1.3 (0.7–2.2)	0.395		1.3 (0.7–2.4)	0.481
35–44	0.9 (0.4–2.3)	0.874		1.1 (0.5–2.7)	0.776		1.1 (0.8–1.7)	0.562		1.9 (1.0–3.4)	0.042		3.7 (2.1–6.3)	<0.001
45–54	1.1 (0.4–3.0)	0.755		1.3 (0.6–3.0)	0.459		1.1 (0.7–1.7)	0.651		2.6 (1.5–4.5)	0.001		5.0 (2.9–8.8)	<0.001
55–64	1.2 (0.4–3.4)	0.767		1.8 (0.9–3.6)	0.112		1.2 (0.8–2.0)	0.372		1.7 (0.9–3.1)	0.089		6.5 (3.6–11.5)	<0.001
≥65	1.9 (0.8–5.2)	0.164		1.8 (0.8–4.1)	0.173		1.3 (0.7–2.3)	0.429		1.4 (0.7–2.7)	0.315		15.8 (7.7–32.2)	<0.001
Area														
Urban	Referent			Referent			Referent			Referent			Referent	
Remote	1.4 (0.8–2.5)	0.212		0.9 (0.5–1.4)	0.531		1.1 (0.6–2.1)	0.801		1.0 (0.6–1.8)	0.940		1.7 (0.9–2.9)	0.067
Rural	1.6 (0.9–3.1)	0.141		0.8 (0.5–1.4)	0.440		1.3 (0.8–2.2)	0.235		0.9 (0.6–1.4)	0.620		0.6 (0.4–0.8)	0.002
Region														
North	Referent			Referent			Referent			Referent			Referent	
Central	1.0 (0.5–2.2)	0.922		0.6 (0.4–0.9)	0.039		0.9 (0.5–1.6)	0.771		1.6 (0.8–3.2)	0.148		4.0 (2.2–7.3)	<0.001
South	1.9 (1.1–3.7)	0.030		0.4 (0.2–0.6)	<0.001		1.1 (0.6–2.0)	0.684		1.7 (1.1–2.6)	0.025		4.0 (2.7–6.0)	<0.001
Clinical symptoms														
Least severe	Referent						†			†			†	
Moderate severe	5.0 (2.8–8.7)	<0.001					†			†			†	
Most severe	13.2 (5.9–29.2)	<0.001					†			†			†	
Access to healthcare														
Good	Referent			Referent						†			†	
Moderate	2.5 (1.4–4.4)	0.002		1.2 (0.8–1.9)	0.343					†			†	
Poor	13.4 (7.0–25.6)	<0.001		1.6 (1.1–2.4)	0.016					†			†	
Behavioral and environmental risks														
Low	Referent			Referent			Referent						†	
Moderate	2.1 (1.1–3.9)	0.017		1.5 (1.1–2.2)	0.015		1.2 (0.8–1.6)	0.336					†	
High	2.9 (1.5–5.6)	0.001		2.4 (1.6–3.6)	<0.001		1.5 (1.0–2.1)	0.029					†	
Socioeconomic status														
High	Referent			Referent			Referent			Referent				
Medium	1.0 (0.6–1.7)	0.992		1.2 (0.9–1.6)	0.326		0.8 (0.6–1.1)	0.172		1.4 (1.1–1.9)	0.020			
Low	1.2 (0.6–2.3)	0.526		2.2 (1.4–3.5)	0.001		1.4 (1.0–2.0)	0.045		2.0 (1.4–2.8)	<0.001			
Bootstrapped AUC	0.90 (0.89–0.92)													

*aOR, adjusted odds ratio; AUC, area under the curve. †Variable was not included as predictor for the respective outcome. Sex, age, area, and region were not an outcome in this structural equation model. Model results were weighted using sampling and lost-to-follow-up weights.

Considering associations between domains and covariates, we found more severe clinical symptoms among participants who were >55 years of age, lived in northern Vietnam, had poor access to healthcare, were more exposed to harmful substances, or had lower SES. Poor access to healthcare was more frequently observed in participants living in rural areas and among those with high behavioral and environmental risks or lower SES. Behavioral and environmental risks were less frequently observed in women, younger participants (15–24 years of age), those living in northern locations, and those with high SES. The bootstrapped AUC result showed that our model could predict the TB status of persons living in Vietnam, assuming the input information is adequate, with a probability of 0.90 (95% CI 0.89–0.92, Table 3).

In the sensitivity analyses, when the clinical domain was excluded in model 1, there was no statistically significant difference in the direct effect of sex nor in the effect of access to healthcare, behavioral and environmental risks, and SES on prevalent TB compared with the primary SEM model (Appendix Table 2). We found no statistically significant difference between the main SEM model and sensitivity analysis in model 2, where the main outcome was replaced by TB status defined by the expert panel (Appendix Table 3). This was also the case for analysis model 3, where all Xpert-positive TB cases with a history of previous TB treatment were excluded from the analysis (Appendix Table 4).

## Discussion

In Vietnam, we witnessed an exceptionally large difference in TB prevalence in relation to sex, with behavioral and environmental risks as the biggest contributors to this disparity: sex determined behavioral and environmental risks leading to TB. Even after accounting for all known associated factors of prevalent TB collected in our data, we found that the odds of having TB were still 3 times higher for men than for women, and this difference remained in our sensitivity analyses. Although we cannot exclude unmeasured confounding, our data suggest that in addition to behavioral and environmental factors, access to healthcare, and symptom presentation, there may be biologic factors that render men more vulnerable to TB than women. Such biologic factors may contribute substantially to the M:F ratio for TB observed in Vietnam and globally.

The behavioral and environmental risks domain, while being strongly associated with sex, only exerted a modest effect on prevalent TB. Of note, in the sex-stratified SEM models, the behavioral and environmental risks domain had a statistically significant effect on prevalent TB among men, but not among women (Appendix Figures 1, 2). This domain consisted both of factors affecting the risk for MTB infection (working indoors, contact with TB patients) and of factors affecting the risk for TB disease progression (smoking, excessive drinking), because we believe that the exact pathophysiologic effect of these factors is unknown and may be multiple and interactive. For example, heavy drinkers may also socialize with more persons in poorly ventilated spaces, thus increasing their risk for MTB infection. In Vietnam, men smoke and drink excessively at an overwhelmingly higher rate compared with women, with a smoking rate of 45.3% vs 1.1% (*16*) and an excessive drinking rate of 44.2% vs 1.2% (*17*). This explains the tremendous influence of sex on the behavioral and environmental risks domain, even when the levels of working indoors and contact with TB patients were similar for both sexes. Also, behavioral and environmental risks were higher among the middle-aged compared with the youngest age group, which is consistent with findings of a survey of tobacco consumption in Vietnam in 2015 (*17*). The effect of this pathway on TB in the sex-stratified SEM models solidifies the evidence that smoking and drinking are major drivers of the difference in TB prevalence between men and women.

Although access to healthcare appeared the strongest predictor of tuberculosis prevalence, we did not find a direct effect of sex on the access to healthcare domain, because men and women in Vietnam tend to have similar access to healthcare. This opposes the findings of other studies, where the gap in access to TB health services is stated to be the main reason behind sex-related differences in the TB burden (*2*,*18*). In Vietnam, a nationwide study in 2002 showed that women have longer TB diagnostic delays than men (5.6 weeks vs 4.4 weeks on average) (*19*), but results from a laboratory study in northern Vietnam suggested that women are more likely than men to have sputum smear examinations (*20*). Our study revealed no significant direct effect of sex on access to healthcare but did demonstrate an indirect effect through exposure. This finding is in line with studies indicating an association between heavy drinking and decreased healthcare utilization (*21*,*22*), because drinkers may be less likely to seek preventive care and primary healthcare than abstainers.

Before 1945, only 10% of the population of Vietnam was literate (*23*); thus, in our study, elderly persons most often had no schooling. We found no difference in SES between men and women, but elderly participants (≥60 years of age) reported a much lower SES than younger participants (15–24 years of age). The SES domain showed only a marginal direct association with TB, but significant indirect associations through the other 3 domains. Studies have shown that persons with low SES are more likely to be uninsured, seek healthcare less often, and have poor-quality healthcare, which may have led some participants to visit medical facilities with more severe clinical symptoms during the survey (*24*,*25*). Persons with low SES also are more likely to smoke and drink alcohol excessively (*26*), which suggest the need for welfare-focused interventions for TB control and prevention.

After controlling for all domains, we still found that men in Vietnam are 3 times more likely to have TB than women. Although other latent factors might have contributed to those odds (e.g., silicosis, nutritional status, illicit drug use, social contact patterns), our findings suggest that a direct biologic effect contributes to this sexual disparity. Although the contribution of some domains might be very specific to the setting of Vietnam, we expect that the residual biologic effect might be the same across settings. Further, we suspect that the M:F ratios observed in other surveys might be underestimated, given that confounders (e.g., with respect to access and exposure) were not addressed. Further research should focus on biologic aspects that might influence TB risk among men and women, for example, innate recognition of pathogens and antimicrobial immune responses. Recent mouse model data showed that estrogen in women boosts the potential of macrophages to kill bacteria that cause pneumonia (*27*). Compared with male mice, female mice also produced more IFNγ, an important cytokine that increases the antibacterial functions of macrophages (*28*). Data also suggest that testosterone is a mediator that inhibits the immune system by inhibiting such proinflammatory factors as TNF-α and nitric oxide and that male castration increases TNF-α secretion in mice (*29*,*30*). Aside from the influence of hormones, genetics might have a critical impact on improving women’s susceptibility to infections like TB since the X chromosome expresses several immune-related genes and immune-associated microRNAs. Women therefore may benefit from having 2 X chromosomes (*31*).

Our SEM analysis included all known and commonly occurring associated factors of prevalent TB that were collected in the PEER questionnaire, but we acknowledge that there may be other factors that were not measured. There also might be selection bias among the case group, since this group consisted of participants with only one positive Xpert test, which may include false-positive Xpert results, causing a slight underestimation of the associations. The prevalence survey used BACTEC MGIT 960 liquid culture (Becton, Dickinson and Company, https://www.bd.com/en-us) to diagnose TB, but the turnaround time was too long to inform selection for the PEER study; therefore, 2 participants selected as controls for the study who had MTB-positive culture results had to be excluded from the analyses. In addition, 83 survey participants who were Xpert-negative but culture-positive were missed being selected to participate in the PEER study. There were also 38 cases who were likely to be Xpert false-positive due to their previous TB treatment history. In the sensitivity analysis that excluded these cases, the SEM model yielded similar results. Another limitation of this study is that gender was not explicitly taken into account in our analysis. Indeed, some factors such as access to care and risk behaviors are likely to be more closely associated with gender than with sex, making the delineation between the 2 concepts in our study not straightforward. Neyman bias might have occurred because of our choice of prevalent TB as the outcome. Using prevalence survey data offers the benefit of minimizing sex bias in reporting. However, prevalence reflects both incidence and duration of disease, and we could not distinguish TB patients with different duration of disease in our analysis. Selection bias also might have occurred in our study, because the proportion of Xpert-positive TB cases found among the preselected controls was much higher than the proportion of TB cases found in the overall TB prevalence survey population. The participation rate among the preselected controls was also higher than that of the TB prevalence survey population (*7*). Like the TB prevalence survey, our analysis involved an undersampling in the youngest age group because the prevalence of TB among young persons in Vietnam was very low, which limited the allocation of participants in this age group (*7*).

In conclusion, we attribute the strong sex difference in TB prevalence found in Vietnam to a complex interplay of factors relating to behavioral and environmental risks, access to healthcare, and clinical manifestations. However, after controlling for all these factors, there remains a direct effect that is likely biological. Further insights from basic and clinical research are needed to explore this biologic difference. Aside from addressing other controllable factors, such as access to healthcare and behavioral and environmental risks, efforts to control TB should include effective strategies focused on men and reducing that specific disease burden.

Appendix Additional information about influence of sex and sex-based disparities on tuberculosis prevalence, Vietnam, 2017–2018.
