# Supplementary material for: Influence of Sex and Sex-Based Disparities on Prevalent Tuberculosis, Vietnam, 2017–2018
Source: Emerg Infect Dis. 2023 May;29(5):967–76. doi: 10.3201/eid2905.221476 (PMC10124636; doi:10.3201/eid2905.221476)
Supplement: Appendix — Additional information about influence of sex and sex-based disparities on tuberculosis prevalence, Vietnam, 2017–2018. [file 22-1476-Techapp-s1.pdf]

Article DOI: http://doi.org/10.3201/eid2905.221476

EID cannot ensure accessibility for supplementary materials supplied by authors. Readers who have difficulty accessing supplementary content should contact the authors for assistance.

Influence of Sex and Sex-Based Disparities on Prevalent Tuberculosis, Vietnam, 2017–2018

Appendix

Appendix Table 1. Measurement of contributions in each domain of the structural equation model.

Category	Coefficient	95% CI	*P*
Clinical symptoms			
Weight loss	1.13	0.63–1.62	<0.001
Fever	1.28	0.71–1.86	<0.001
Night sweats	1.98	0.74–3.22	0.002
Cough	1		
Access to healthcare			
First healthcare facility visited	2.30	0.8–3.8	0.002
Health insurance status	0.46	0.17–0.74	0.002
HIV status	0.87	0.47–1.26	<0.001
Chest x-ray taken in the past	0.98	0.53–1.42	<0.001
Distance from home to the nearest hospital	1		
Behavioral and environmental risks			
Work indoor	0.50	0.30–0.70	<0.001
Miner	−0.19	−1.07–0.70	0.681
Contacted with TB patients	0.55	0.29–0.81	<0.001
Diabetes	0.33	−0.10–0.69	0.067
Excessive drinking	0.86	0.34–1.36	0.001
Passive smoking	3.26	1.20–5.35	0.002
Amount of pack-year	−0.20	−0.52–0.12	0.221
Smoking	1		
Socio-economic status			
Household assets	0.45	0.23–0.68	<0.001
Occupation	0.53	0.27–0.79	<0.001
Marital status	0.07	−0.13–0.47	0.104
Educational level	1		

Confirmatory Factor Analysis results, where each domain was estimated separately as a latent variable that gives rise to the observed underlying measures of the respective domain. The strongest factor loading of a domain is the best measure of that domain.
Clinical symptoms: Weight loss: Yes – No; Fever: Yes – No; Night sweats: Yes – No; Cough: Yes- No.
Access to healthcare: First healthcare facility visited: Public sector facility – Private sector facility; Health insurance status: Insured – No insurance; HIV status: HIV negative – HIV unknown; Chest x-ray taken in the past: Yes – No; Distance from home to the nearest hospital: Near (less than 15 min of traveling) – Far (more than 15 min of traveling).
Behavioral and environmental risks: Work indoor: Indoor – Outdoor – Both; Miner: Yes – No; Contacted with TB patients: No contacting – At work – At home – Other places – Unknown.; Diabetes: Yes – No; Excessive drinking: Yes (Having at least one occasion of drinking at least 5 drinks for men and 4 drinks for women in the last 30 d) – No; Passive smoking: Yes (Having another person smoking in your environment in the last 30 d) – No; Amount of pack-year: First tertile (<10 pack-years) – Second tertile (10–20 pack-years) – Third tertile (>20 pack-years); Smoking: Non-smoker – Ex-smoker – Current smoker.
Socio-economic status: Household assets: Rich – Medium – Poor; Occupation: State officials – Non-governmental organizations officials – Self-employed – Unemployed; Marital status: Single (including never married, divorced, separated, and widowed) – Married; Educational level: Undergraduate/Postgraduate – Highschool – Secondary school – Primary school – No schooling.

Appendix Table 2. Sensitivity analysis 1: Structural Equation Model full estimation results of the relationships between sex, behavioral and environmental risks, access to healthcare, socioeconomic status, and Xpert(+) prevalent TB.

	**Xpert (+) prevalent TB**			**Access to care**			**Behavioral and environmental risks**			**Socioeconomic status**		
**OUTCOME:**	aOR*	95% CI	*P*	aOR*	95% CI	*P*	aOR*	95% CI	*P*	aOR*	95% CI	*P*
Sex: Males →	2.7	1.4–4.3	<0.001	1.4	1.1–1.7	0.005	7.7	6.0–9.8	<0.001	0.8	0.6–1.0	0.055
Age groups												
15–24 y old		Reference			Reference			Reference			Reference	
25–34 y old	1.1	0.4–3.1	0.858	1.1	0.7–1.7	0.661	1.3	0.7–2.2	0.395	1.3	0.5–1.4	0.481
35–44 y old	0.8	0.3–2.3	0.723	1.1	0.8–1.7	0.562	1.9	1.0–3.4	0.042	3.7	2.1–6.3	<0.001
45–54 y old	1.1	0.4–3.2	0.830	1.1	0.7–1.7	0.651	2.6	1.5–4.5	0.001	5.0	2.9–8.8	<0.001
55–64 y old	1.3	0.5–3.9	0.598	1.2	0.8–2.0	0.372	1.7	0.9–3.1	0.089	6.5	3.6–11.5	<0.001
≥65 y old	2.1	0.7–5.7	0.170	1.3	0.7–2.3	0.429	1.4	0.7–2.7	0.315	15.8	7.1–32.2	<0.001
Area												
Urban		Reference			Reference			Reference			Reference	
Remote	1.1	0.6–2.2	0.741	1.1	0.6–2.1	0.801	1.0	0.6–1.8	0.940	2.0	1.1–3.8	0.034
Rural	1.3	0.8–2.3	0.316	1.3	0.9–2.2	0.235	0.9	0.6–1.4	0.620	0.4	0.2–0.8	0.005
Region												
North		Reference			Reference			Reference			Reference	
Central	0.9	0.4–1.7	0.687	0.9	0.5–1.6	0.771	1.6	0.8–3.2	0.148	5.2	2.4–11.4	<0.001
South	1.4	0.7–2.5	0.319	1.1	0.6–2.0	0.684	1.7	1.1–2.6	0.025	4.8	2.8–8.2	<0.001
Access to care												
Good access		Reference						¶			¶	
Moderate access	2.4	1.3–4.4	0.007					¶			¶	
Poor access	11.2	5.7–22.1	<0.001					¶			¶	
Behavioral and environmental risks												
Low risk		Reference			Reference						¶	
Moderate risk	2.5	1.5–4.3	<0.001	1.2	0.9–1.6	0.336					¶	
High risk	3.7	2.0–6.7	<0.001	1.5	1.0–2.1	0.029					¶	
Socioeconomic status												
High		Reference			Reference			Reference				
Medium	1.6	1.0–2.7	0.059	0.2	0.6–1.1	0.172	1.4	1.1–1.9	0.020			
Low	1.8	0.9–3.4	0.063	1.4	1.0–2.0	0.045	2.0	1.4–2.8	<0.001			
Bootstrapped AUC	0.86	0.83–0.88										

*Adjusted odds ratio (aOR). ¶ Variable was not included as predictor for the respective outcome. Sex, age, area, and region were not an outcome in this Structural Equation Model. Model results were weighted using sampling and lost to follow up weights.

Appendix Table 3. Sensitivity analysis 2: Structural Equation Model full estimation results of the relationships between sex, clinical symptoms, behavioral and environmental risks, access to healthcare, socioeconomic status, and panel defined prevalent TB.

	Panel-defined prevalent TB			Clinical symptoms			Access to care			Behavioral and environmental risks			Socioeconomic status		
**OUTCOME:**	aOR*	95% CI	*P*	aOR*	95% CI	*P*	aOR*	95% CI	*P*	aOR*	95% CI	*P*	aOR*	95% CI	*P*
Sex: Males →	3.5	1.9–6.5	<0.001	1.2	0.9–1.7	0.281	1.4	1.1–1.7	0.005	7.7	6.0–9.8	<0.001	0.8	0.6–1.0	0.057
Age groups															
15–24 y old		Reference			Reference			Reference			Reference			Reference	
25–34 y old	1.1	0.5–2.6	0.819	1.2	0.5–2.4	0.706	1.1	0.7–1.7	0.611	1.3	0.8–2.3	0.395	1.3	0.7–2.4	0.445
35–44 y old	0.9	0.4–2.0	0.703	1.1	0.5–2.7	0.776	1.1	0.8–1.7	0.562	1.9	1.1–3.5	0.042	3.7	2.1–6.3	<0.001
45–54 y old	1.1	0.5–2.6	0.816	1.3	0.6–3.0	0.459	1.1	0.7–1.7	0.651	2.6	1.5–4.6	0.001	5.0	2.9–8.8	<0.001
55–64 y old	1.2	0.5–3.1	0.678	1.8	0.9–3.6	0.122	1.2	0.8–2.0	0.372	1.7	0.9–3.0	0.089	6.4	3.6–11.5	<0.001
≥65 y old	1.8	0.8–4.4	0.171	1.8	0.8–4.1	0.173	1.3	0.7–2.2	0.429	1.4	0.7–2.8	0.315	15.7	7.7–32.2	<0.001
Area															
Urban		Reference			Reference			Reference			Reference			Reference	
Remote	1.5	0.9–2.5	0.165	0.9	0.5–1.4	0.531	1.1	0.6–2.1	0.801	1.0	0.5–1.8	0.968	2.0	1.1–3.8	0.034
Rural	1.7	0.9–3.4	0.111	0.8	0.5–1.3	0.440	1.3	0.8–2.1	0.235	0.9	0.6–1.4	0.709	0.4	0.2–0.8	0.005
Region															
North		Reference			Reference			Reference			Reference			Reference	
Central	1.2	0.5–2.6	0.650	0.6	0.4–0.9	0.039	0.9	0.5–1.6	0.771	1.6	0.8–3.2	0.148	5.2	2.4–11.4	<0.001
South	2.2	1.2–4.2	0.016	0.4	0.2–0.6	<0.001	1.1	0.6–2.0	0.684	1.7	1.0–2.6	0.025	4.8	2.8–8.2	<0.001
Clinical symptoms															
Least severe		Reference						¶			¶			¶	
Moderate severe	5.8	3.4–10.0	<0.001					¶			¶			¶	
Most severe	8.5	4.0–18.2	<0.001					¶			¶			¶	
Access to care															
Good access		Reference			Reference						¶			¶	
Moderate access	3.2	1.6–6.5	0.002	1.2	0.8–1.9	0.343					¶			¶	
Poor access	12.4	6.3–24.4	<0.001	1.6	1.1–2.4	0.016					¶			¶	
Behavioral and environmental risks															
Low risk		Reference			Reference			Reference						¶	
Moderate risk	2.0	1.1–3.6	0.023	1.5	1.1–2.2	0.015	1.2	0.9–1.6	0.336					¶	
High risk	1.7	1.0–2.9	0.062	2.4	1.6–3.6	<0.001	1.5	1.0–2.1	0.029					¶	
Socioeconomic status															
High		Reference			Reference			Reference			Reference				
Medium	1.2	0.6–2.1	0.612	1.2	0.9–1.6	0.326	0.8	0.6–1.1	0.172	1.4	1.1–1.9	0.020			
Low	1.4	0.7–2.8	0.296	2.2	1.4–3.5	0.001	1.4	1.0–2.0	0.045	2.0	1.4–2.8	<0.001			
Bootstrapped AUC	0.89	0.88–0.92													

*Adjusted odds ratio (aOR).

¶ Variable was not included as predictor for the respective outcome. Sex, age, area, and region were not an outcome in this Structural Equation Model. Model results were weighted using sampling and lost to follow up weights.

Appendix Table 4. Sensitivity analysis 3: Structural Equation Model full estimation results of the relationships between sex, clinical symptoms, behavioral and environmental risks, access to healthcare, socioeconomic status, and Xpert(+) prevalent TB, excluding 38 cases who had a previous TB treatment history.

	Xpert (+) prevalent TB			Clinical symptoms			Access to care			Behavioral and environmental risks			Socioeconomic status		
**OUTCOME:**	aOR*	95% CI	*P*	aOR*	95% CI	*P*	aOR*	95% CI	*P*	aOR*	95% CI	*P*	aOR*	95% CI	*P*
Sex: Males →	3.1	1.7–5.6	<0.001	1.2	0.8–1.7	0.324	1.4	1.1–1.7	0.006	7.5	5.8–9.7	<0.001	0.8	0.6–1.1	0.066
Age groups															
15–24 y old		Reference			Reference			Reference			Reference			Reference	
25–34 y old	1.1	0.4–2.7	0.685	1.2	0.6–2.5	0.665	1.1	0.7–1.7	0.639	1.3	0.7–2.2	0.418	1.3	0.7–2.4	0.428

OUTCOME:	Xpert (+) prevalent TB			Clinical symptoms			Access to care			Behavioral and environmental risks			Socioeconomic status		
	aOR*	95% CI	*P*	aOR*	95% CI	*P*	aOR*	95% CI	*P*	aOR*	95% CI	*P*	aOR*	95% CI	*P*
35–44 y old	0.7	0.3–1.7	0.441	1.2	0.5–2.8	0.736	1.1	0.7–1.6	0.707	1.9	0.9–3.2	0.055	3.6	2.1–6.3	<0.001
45–54 y old	1.0	0.4–2.5	0.953	1.2	0.6–2.9	0.590	1.1	0.7–1.6	0.796	2.6	1.4–4.3	0.002	4.9	2.8–8.8	<0.001
55–64 y old	1.1	0.4–3.1	0.877	1.8	0.9–3.7	0.118	1.2	0.8–2.0	0.399	1.7	0.9–3.0	0.108	6.5	3.7–11.6	<0.001
≥65 y old	1.7	0.7–4.4	0.284	1.7	0.8–4.0	0.191	1.2	0.7–2.2	0.498	1.4	0.7–2.6	0.375	15.2	7.5–30.7	<0.001
Area															
Urban		Reference			Reference			Reference			Reference			Reference	
Remote	1.5	0.8–2.7	0.205	0.8	0.5–1.3	0.419	1.1	0.6–2.1	0.785	1.0	0.5–1.8	0.971	1.6	0.9–2.6	0.085
Rural	1.7	0.9–3.3	0.114	0.8	0.5–1.4	0.480	1.3	0.9–2.2	0.191	0.9	0.6–1.4	0.662	0.5	0.4–0.8	0.001
Region															
North		Reference			Reference			Reference			Reference			Reference	
Central	1.0	045–2.3	0.981	0.6	0.4–0.9	0.038	0.9	0.5–1.6	0.693	1.6	0.8–3.0	0.177	4.0	2.3–7.2	<0.001
South	2.1	1.1–4.1	0.020	0.4	0.2–0.6	<0.001	1.1	0.6–2.0	0.731	1.7	1.0–2.5	0.024	4.0	2.7–5.9	<0.001
Clinical symptoms															
Least severe		Reference						¶			¶			¶	
Moderate severe	5.6	3.1–10.0	<0.001					¶			¶			¶	
Most severe	14.5	6.2–34.1	<0.001					¶			¶			¶	
Access to care															
Good access		Reference			Reference						¶			¶	
Moderate access	2.1	1.1–3.9	0.018	1.2	0.8–1.8	0.484					¶			¶	
Poor access	11.7	6.0–22.3	<0.001	1.7	1.1–2.5	0.015					¶			¶	
Behavioral and environmental risks															
Low risk		Reference			Reference			Reference						¶	
Moderate risk	2.0	1.0–3.9	0.037	1.5	1.0–2.1	0.030	1.1	0.8–1.5	0.436					¶	
High risk	2.7	1.3–5.5	0.006	2.4	1.6–3.6	<0.001	1.5	1.0–2.1	0.028					¶	
Socioeconomic status															
High		Reference			Reference			Reference			Reference				
Medium	1.1	0.6–1.9	0.792	1.1	0.8–1.5	0.432	0.9	0.7–1.3	0.656	1.5	1.1–2.1	0.013			
Low	1.2	0.6–2.3	0.667	2.1	1.4–3.4	0.001	1.6	1.2–2.3	0.004	2.1	1.5–3.0	<0.001			
Bootstrapped AUC	0.90	0.89–0.92													

*Adjusted odds ratio (aOR). ¶ Variable was not included as predictor for the respective outcome. Sex, age, area, and region were not an outcome in this Structural Equation Model. Model results were weighted using sampling and lost to follow up weights.

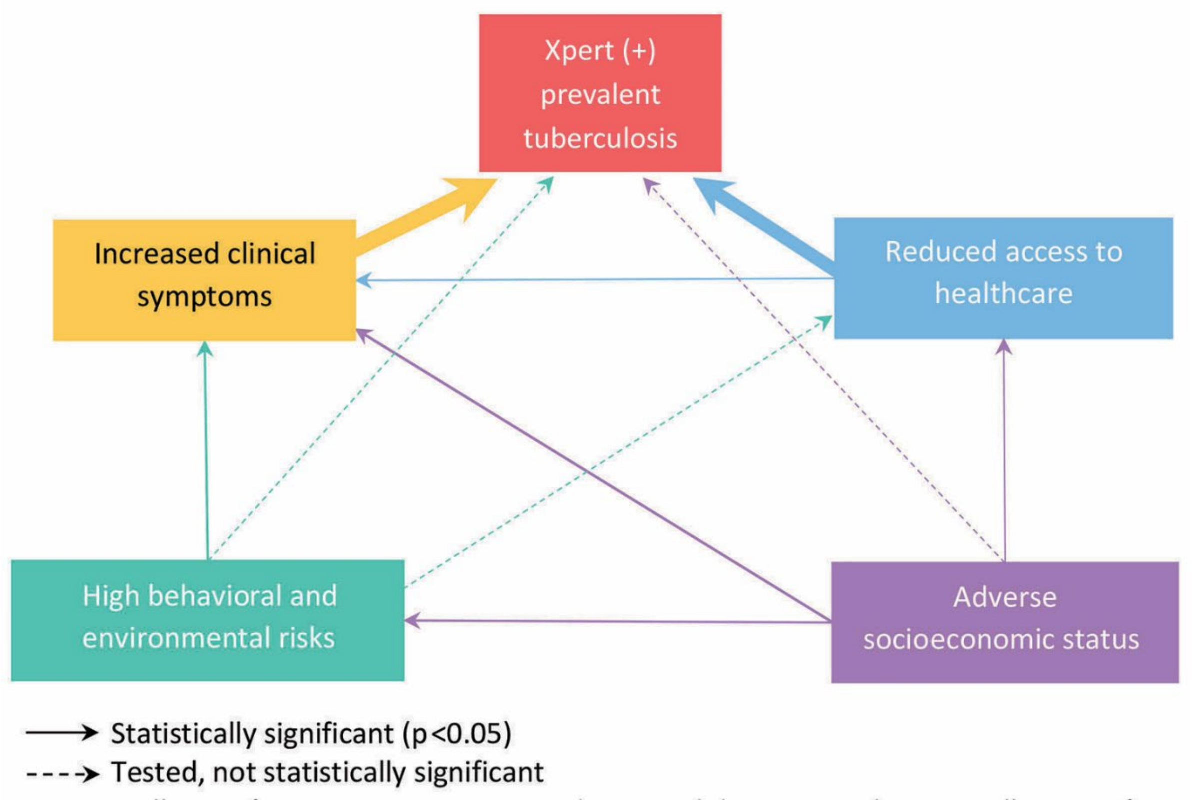

Appendix Figure 1. Structural equation model with pathway analysis between tuberculosis and other domains among women. Statistically significant associations are indicated by the solid arrows and statistically insignificant associations by the dashed arrows. For the significant associations, the arrow thickness corresponds to the effect size. Each outcome was adjusted for age, area, and region. Model results were weighted using sampling and lost-to-follow-up weights. Bootstrapped area under the curve (1,000 replications) was 0.83 (95% CI 0.80–0.86).

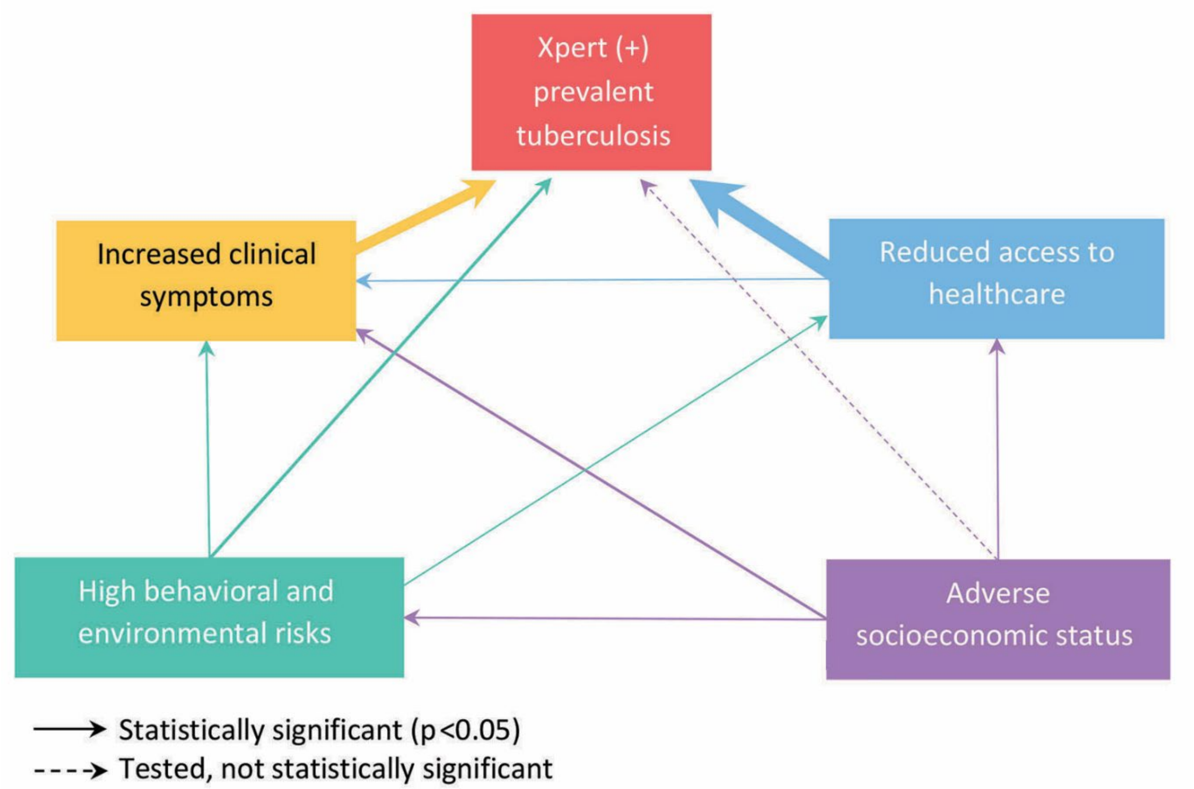

Appendix Figure 2. Structure equation model with pathway analysis between tuberculosis and other domains among men. Statistically significant associations are indicated by the solid arrows and statistically insignificant associations by the dashed arrows. For the significant associations, the arrow thickness corresponds to the effect size. Each outcome was adjusted for age, area, and region. Model results were weighted using sampling and lost-to-follow-up weights. Bootstrapped area under the curve (1,000 replications) was 0.89 (95% CI 0.87–0.91).
